# Aging-related decline in the liver and brain is accelerated by refined diet consumption

**DOI:** 10.1007/s11357-025-01897-y

**Published:** 2025-09-26

**Authors:** Franziska Kromm, Haktan Övül Bozkir, Annette Brandt, Timur Yergaliyev, Amélia Camarinha-Silva, Ina Bergheim

**Affiliations:** 1 Department of Nutritional Sciences, Molecular Nutritional Science, University of Vienna, Josef-Holaubek-Platz 2 (UZA II), Vienna, 1090 Austria; 2 Institute of Animal Science, University of Hohenheim, Emil-Wolff-Str. 6-10, Stuttgart, 70593 Germany

**Keywords:** Dietary fiber, Aging, Cognition, Cellulose, β-glucan

## Abstract

Studies suggest that diets rich in highly/ultra-processed foods may contribute to the development of diseases. In rodents, the intake of refined (purified) diets has also been associated with the development of various metabolic diseases. Here, performing two experiments, we assess the impact of a refined diet with and without fiber enrichment on aging related health decline in mice. Experiment 1: Male C57BL/6J mice were fed standard chow or refined diet until the age of 86 weeks. Experiment 2: Male C57BL/6J mice fed a refined diet until showing signs of aging related intestinal barrier dysfunction were fed a fiber enriched refined diet (7.5% oat β-glucan or 7.5% cellulose) for 18 weeks. Cognition was assessed at the beginning and end of the dietary fiber intervention, while glucose tolerance was determined at the end of each experiment. Markers of senescence, liver damage, neuroinflammation, intestinal barrier function, and microbiota composition were assessed. Refined diet-fed mice showed higher markers of senescence in plasma and aging-associated liver decline, impaired glucose metabolism and cognitive decline compared to standard diet-fed mice. These changes were associated with differences in intestinal microbiota composition and higher portal bacterial endotoxin levels. Enriching the refined diet with the fibers decelerated the aging-associated cognitive impairments while not affecting aging-related liver decline, insulin resistance or markers of intestinal barrier function. In conclusion, these data suggest that while decelerating aging-related cognitive decline, fiber fortification of a refined diet may not attenuate the diet-induced acceleration of aging related decline in other organs in mice.

**Supplementary Information:**

The online version contains supplementary material available at 10.1007/s11357-025-01897-y.

## Introduction

With the increasing industrialization of food production and novel technologies for food processing, the availability and consumption of convenient, high- or even ultra-processed refined foods has increased significantly throughout the last decades. Highly- and ultra-processed foods are often calorie dense foods lacking nutritional compounds, e.g., secondary plant compounds but also fiber while being rich in refined carbohydrates like sucrose or fructose. Results of recent studies suggest that diets rich in highly- and ultra-processed foods may be critical contributors to the development of metabolic diseases but may also affect cognition [1, 2]. However, data obtained in epidemiological studies are conflicting. Also, there is an ongoing discussion about whether the processing of foods leads to an increased health risk or if ultra-processed food consumption is simply a marker of poor diet quality [2]. Studies in animal models suggest that refined diets, compared to standard chow, are related to increased neuroinflammatory signaling [3] and an accelerated onset of diabetes and liver steatosis [4]. This has also been linked to changes in intestinal microbiota composition [4, 5]. Indeed, standard chows used in animal husbandry are grain-based diets containing natural ingredients like ground corn, oat and wheat often supplemented with a mix of soluble and insoluble fiber (for overview [6]). Employing this approach standard chows thereby provid additional `nutritive´ compounds also found in low processed foods in human diets. If and how the intake of a refined diet affects the natural course of aging and the related decline observed in various organs and tissues has yet neither been fully clarified in rodents nor humans.

Dietary fiber has been suggested to reduce the risk for the development of metabolic diseases but also to promote ‘healthy’ aging [7]. Alterations of intestinal homeostasis are discussed as critical in aging related health decline [8]. Results of previous studies of our own group and data from other research groups further indicate that aging in mouse models and humans is associated with an increased translocation of bacterial endotoxins and an impaired intestinal barrier function [9–13]. Moreover, enriching the diet with dietary fibers has been shown to improve intestinal barrier function and decrease bacterial endotoxin levels in blood in settings of diet induced metabolic diseases ([14] and for overview [15, 16]). Also, it has been shown that the addition of purified fiber (e.g., cellulose, lignocellulose, pectin, and inulin) to a refined diet may at least in part dampen the metabolic effects of a refined diet in mice [5]. If similar effects are also found with respect to aging related decline when purified fibers are added to the diet of aging mice has not yet been determined. Indeed, studies have demonstrated that various types of dietary fiber e.g., soluble or insoluble fiber, as well as fibers derived from different food sources or those classified as viscous or non-viscous, can have distinct health benefits [17]. Studies further suggest that soluble, highly viscous fibers, such as gel-forming, fermentable β-glucans from oats bear potential to reduce blood cholesterol and the risk of coronary heart disease [18]. In contrast, insoluble fibers such as cellulose, which have a low fermentation rate and are able to increase stool moisture content and bulk [19] might be able to induce cellular and molecular anti-inflammatory mechanisms [20].

Here, we investigated the impact of a refined low-fat diet on aging related decline. In particular, we explored effects on aging related changes in liver, brain, and cognition as well as intestine. Furthermore, we assessed the effect of the addition of purified fibers (β-glucan derived from oat and cellulose, respectively) on alterations observed.

## Material and methods

### Mice experiments

All experiments involving animals were approved by the local institutional animal care and use committee (Federal Ministry Republic of Austria Education, Science and Research, Vienna, Austria, 2021–0.464.116, 2022–0.527.442). Male C57BL/6J mice (The Jackson Laboratory, USA) were bred and aged in a specific-pathogen-free barrier facility accredited by the Association for Assessment and Accreditation of Laboratory Animal Care located at the University of Vienna. Until the age of 6 months all mice received standard chow (V1534-300, Ssniff GmbH, Germany, based on wheat and barley as well as soy derived products, minerals, oat hulls/bran, sugar beet pulp). Mice were housed under controlled conditions in individually ventilated cages (3–5/cage) and had free access to food and tap water at all times. All mouse experiments were performed in the same animal facility and same room. In a first experiment, mice (*n* = 8–10/group) fed standard chow were compared to mice fed the refined diet starting at the age of 6 months (~ 26 weeks). The refined diet being a modified AIN93G diet composed of highly refined ingredients, such as casein, corn starch, maltodextrin, sucrose, soybean oil and containing 5% cellulose as only fiber source (Ssniff GmbH, Germany, details see Supplemental Table 1) was fed to mice until they reached 86 weeks of age. Sample size was based on power calculations and the previously shown increase in bacterial endotoxin concentrations in mice during aging [11] assuming an alpha error = 0.05, beta error = 0.1 and effect strength of 1.43. In a second experiment, mice aged 6 months were fed the refined diet until the first signs of intestinal barrier dysfunction, specifically until significant increases in bacterial endotoxin levels in peripheral blood were detected (68 weeks of age). Mice were then switched to a refined diet containing 7.5% β-glucan derived from oats (Garuda international, Inc., USA) or 7.5% cellulose (Arbocel®, JRS, Germany) as only source of fiber for 18 weeks. With respect to the 3R concept, mice from the second set of experiments were compared to mice fed the refined diet containing 5% cellulose as only fiber source from the first set of experiments. Composition of diet is shown in Supplemental Table 1. Bacterial endotoxin was assessed monthly starting at the age of 62 weeks in blood obtained from *vena fascialis* using the LAL assay as detailed before [9]. Before being switched to the fiber enriched diets, working memory was assessed in a spatial novelty preference Y-maze paradigm as described before [10]. In brief, after spending 5 min in a Y-maze with two of three arms open, the mouse was placed in a holding cage for 1 min following the Y-maze with now all three arms open for another 2 min. Time spent in the new arm was assessed. Body weight was assessed weekly. The Y-Maze test was repeated two weeks before the end of the experiment. Moreover, a glucose tolerance test (GTT) was performed one week before sacrifice. In brief, after fasting mice for 6 h, 2 g/kg BW glucose was injected i.p. and glucose concentration was assessed in blood from tail vein after 15, 30, 60, 90, and 120 min. At the end of the experiment, animals were anaesthetized with a mixture of ketamine/xylazine (100 mg ketamine/kg BW; 16 mg xylazine/kg BW, i.p. injection) and mice were killed by cervical dislocation. Blood from portal vein, liver, intestinal and brain tissue samples were collected for further analysis.

**Table 1:** Effect of an extended intake of a refined or standard diet on body and liver weight in aging male C57BL/6 mice

	Groups	
	Standard chow	Refined diet
Absolute body weight (g)	36.8 ± 1.3	35.9 ± 1.1
Liver weight (g)	2.0 ± 0.1	1.6 ± 0.1*
Liver: body weight ratio (%)	5.4 ± 0.1	4.6 ± 0.1*

Data are presented with means ± SEM. *p < 0.05. *n* = 8–10

### Liver damage

Liver tissue sections (4 µm) from embedded tissue were stained with hematoxylin and eosin (H&E, Sigma-Aldrich, Germany) and evaluated by using the NAFLD Activity Score as described before [21]. Neutrophil granulocytes were stained and counted in liver tissue sections using a commercially available kit (Naphthol AS-D Chloroacetate (Specific Esterase) Kit; Sigma-Aldrich, Germany) as detailed before [22]. Sirius red staining in liver sections was performed as previously described [12]. Triglyceride concentration in liver homogenates was determined as previously described [22].

### Ex vivo everted gut sac

In order to assess if the different diets had different effects on intestinal barrier function, permeability of xylose in everted tissue sacs of small and large intestinal tissue was assessed as described in detail before [23]. In brief, everted gut sacs were filled with 1 × Krebs–Henseleit-bicarbonate buffer, consisting of 0.2% (w/v) bovine serum albumin (KRH buffer) and incubated for 5 min in gassed KRH buffer containing 0.1% (w/v) D-xylose. Xylose concentration was determined within the everted gut sacs, as described before [24] and calculated in mmol/L.

### Endotoxin measurement

Concentration of endotoxin in plasma from portal vein was assessed as previously described by using a LAL-assay (Charles River, USA) [9].

### Western blot analysis

To determine markers of senescence and inflammation, total plasma protein was separated on a polyacrylamide gel and transferred to polyvinylidene difluoride membranes (Bio-Rad Laboratories, USA) as described previously [10]. In brief, membranes were blocked with either bovine serum albumin (BSA) or non-fat dry milk powder and were incubated with primary antibodies for fibrinogen, C-reactive protein (CRP) and interleukin 6 (IL6) (CRP, fibrinogen, IL6, Santa Cruz, USA), and respective secondary antibodies. Band intensities were determined using Super Signal West Dura kit (Thermo Fisher Scientific, USA). Densitometric analysis of bands was performed using ImageLab Software V 6.1.0 (Bio-Rad, USA) and normalized to ponceau.

### Immunohistochemical staining

To detect ionized calcium-binding adapter molecule 1 (IBA1) protein (proteintech, USA) in paraffin embedded brain sections (4 µm) of the right hemisphere, sections were deparaffinized and rehydrated and demasked with Tris EDTA buffer (10 mM Tris base, 1 mM EDTA, 0.05% Tween20, pH9), sections were blocked with peroxidase blocking solution for 10 min (Agilent Dako, USA) and incubated in 5% goat serum in TBS for 1 h. After an incubation with the respective primary antibody, sections were incubated with peroxidase-linked secondary antibody and antibody binding was detected by diamino-benzidine (Agilent Dako, USA). The intensities of the IBA1 protein staining of each microscopic field were evaluated using an analysis system (Leica Applications Suite V4.5, Leica, Germany) integrated in a microscope (Leica DM6 B, Leica, Germany) [10].

### DNA libraries for microbiota analysis

For microbiome analyses, DNA was extracted from colon samples using FastDNA™ Spin Kit for soil (MP Biomedicals, Santa Ana, CA, USA), and then quantified with NanoDrop 2000 spectrophotometer (Thermo Scientific, Waltham, MA, United States). Bacterial communities were targeted by V1–V2 primers [25]. Unique barcodes (6-nt long sequences attached to the forward primers) were used in combination with index adapters (linked to the reverse primers) for sample identification. Three rounds of PCR were used to create the sequencing library, as previously described [12].

### Statistical analysis and bioinformatics

PRISM (Version 7.03, GraphPad Software, Inc.) was used for statistical analysis. Outliers were determined with Grubb´s test. Data were log-transformed if Bartlett´s test was unequal. A one-way analysis of variance (ANOVA) was used to determine statistical differences between three groups followed by Tukey´s post hoc test. Two groups were compared by unpaired or paired *t*-test. All data are presented as means ± standard error of means (SEM). *P* < 0.05 was defined to be significant.

After 16S rRNA gene libraries sequencing, raw reads were demultiplexed by Sabre (https://github.com/najoshi/sabre). Bioinformatic analyses were performed within the Qiime2 pipeline [26]. In short, the q2-cutadapt plugin [27] was used for trimming primers and adapters, and the q2-dada2 [28] for denoising and merging paired reads. Obtained amplicon sequence variants (ASV) were annotated with VSEARCH-based consensus [29] and pre-fitted sklearn-based classifiers [30] against the Silva database (v138.2, 16S 99%) [31], prepared by RESCRIPt [32]. Shannon’s entropy and Faith’s phylogenetic diversity (Faith’s PD) were calculated to measure alpha diversity, and for beta diversity the phylogenetic robust Aitchison distances (phyRPCA) [33] were calculated. Statistical analyses of alpha diversity indices were performed using the ANOVA test and beta diversity distances were compared using the Adonis test (999 permutations). The formula “Group + Cage” was used for both ANOVA and Adonis tests to account for the cage effect. All P-values obtained from multiple comparisons were adjusted using the Benjamini–Hochberg procedure. Absolute taxa counts (at genus level) with relative abundance ≥ 1% and prevalence ≥ 10% were used for the differential abundance test by the Ancom-BC.

##  Results

### Effect of an extended intake of a refined diet on markers of senescence, glucose tolerance, and liver damage as well as cognitive alterations in aged C57BL/6 mice

**Fig. 1:**
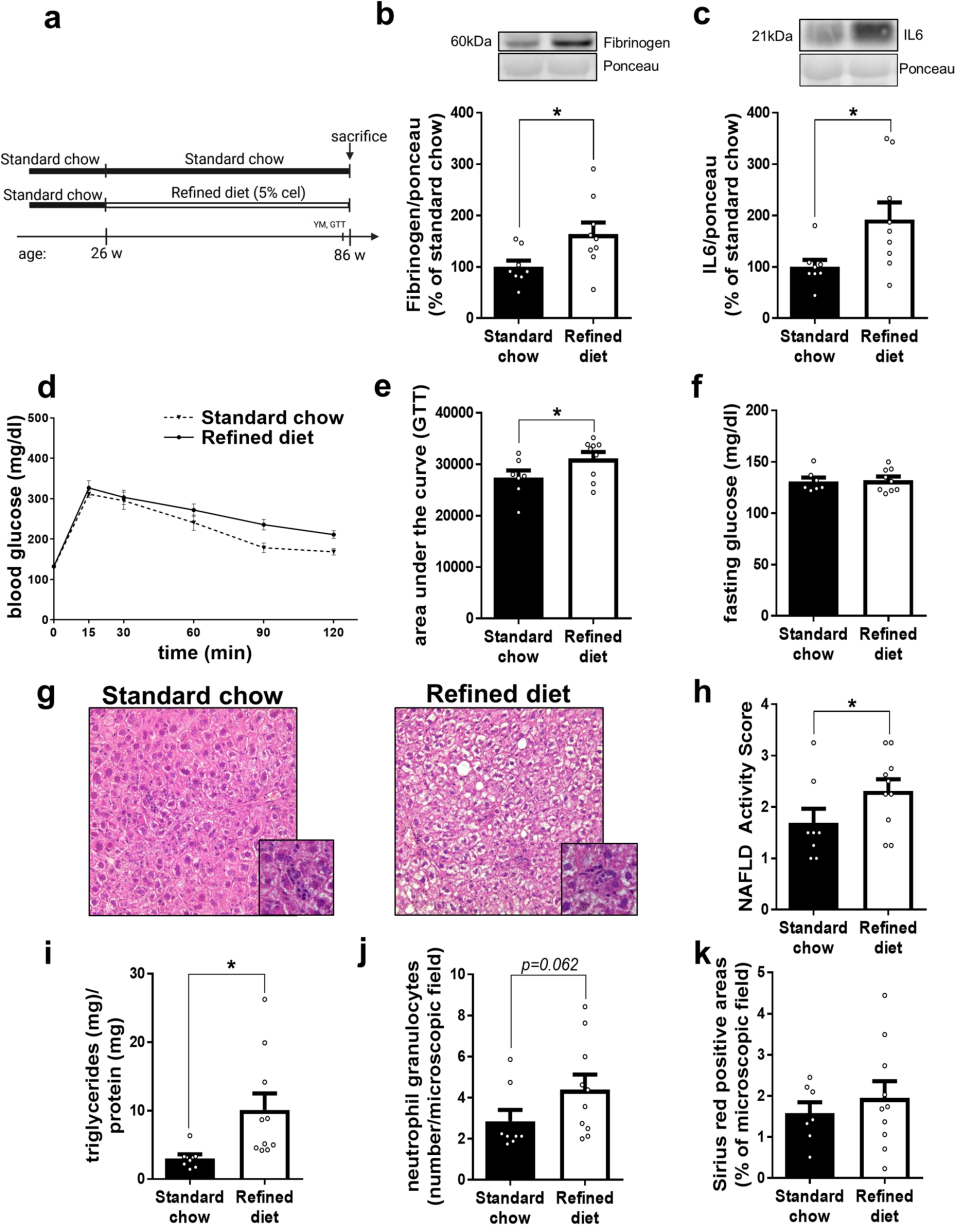
Effect of an extended intake of a refined or standard diet on markers of senescence, glucose metabolism and liver damage and in aging male C57BL/6 mice. (**a**) Study design. Densitometric analysis of (**b**) fibrinogen (**c**) and interleukin 6 (IL6) western blot bands normalized to ponceau in plasma and representative blots. (**d**) Glucose concentration during glucose tolerance test (GTT) and (**e**) area under the curve of GTT as well as (**f**) fasting blood glucose. (**g**) Representative pictures of hematoxylin and eosin-stained liver (200 ×, 630 ×) and (**h**) evaluation via NAFLD activity score, (**i**) triglyceride concentration in liver homogenates, (**j**) number of neutrophil granulocytes and (**k**) Sirius red positive stained areas in liver sections of aging mice receiving a standard chow or refined diet. w – weeks, YM – Y-Maze. Data are presented with means ± SEM. *p < 0.05. *n* = 7–10

To determine, if an extended intake of a refined diet affects biological aging (Study design see Fig. 1a), we first determined fibrinogen and IL6 protein levels in plasma of old mice. Compared to standard chow fed mice, mice that had been fed the refined diet showed higher levels of fibrinogen and IL6 protein in plasma (Fig. 1b–c). The more progressed senescence in refined diet fed mice was related to significantly higher glucose concentrations when performing GTT in these mice compared to standard chow fed mice (Fig. 1d–e). In contrast, fasting blood glucose levels were similar between groups (Fig. 1f). Moreover, despite showing no differences in body weight, but a reduction in liver: body weight ratio (Table 1), aging-related liver degeneration e.g., fat accumulation and inflammation, was more progressed in refined diet fed mice than in standard chow fed mice. Indeed, NAFLD activity score, triglyceride concentration as well as number of neutrophil granulocytes were higher in mice fed the refined diet compared to mice fed a standard chow (NAFLD activity score and triglycerides: *p* < 0.05, neutrophil granulocytes: *p* = 0.062) (Fig. 1g–j). However, fibrosis, as determined by % of Sirius red stained area, was in an early stage and similar between groups (Fig. 1k).

**Fig. 2:**
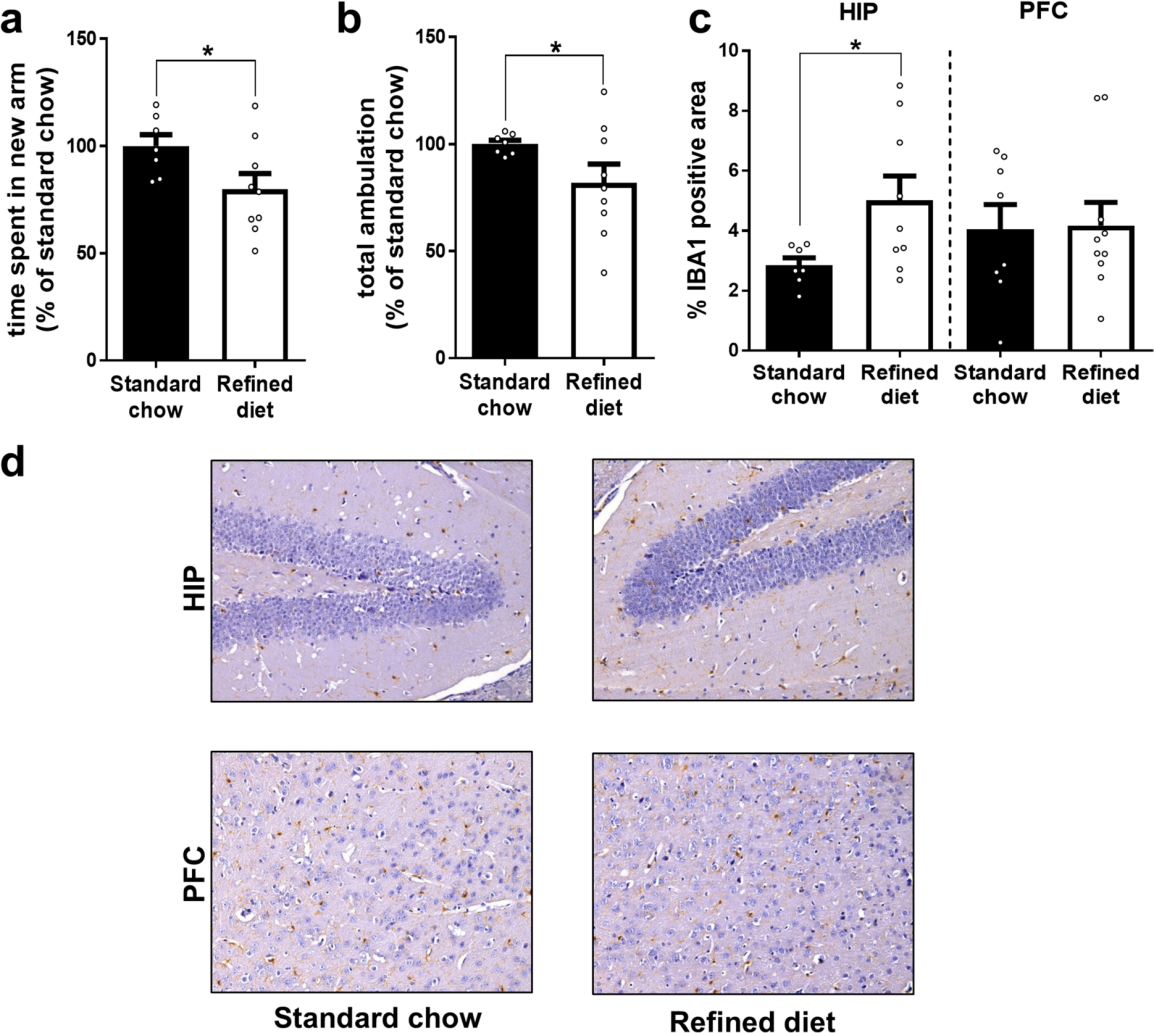
Effect of an extended intake of a refined or standard diet on cognitive alterations and markers of neuroinflammation in aging male C57BL/6 mice (**a**) Time spent in new arm and (**b**) total ambulation in Y-Maze, (**c**) ionized calcium-binding adapter molecule 1 (IBA1) positive stained area in brain sections and (**d**) representative pictures of hippocampus (HIP) (200 ×) as well as prefrontal cortex (PFC) (200 ×) of aging mice receiving a standard chow or refined diet. Data are presented with means ± SEM. *p < 0.05. *n* = 7–10

Short-term spatial memory was significantly lower in aged mice fed the refined diet compared to mice fed standard chow (Fig. 2a). Specifically, mice fed the refined diet spent significantly less time in the “new arm” of the Y-maze than those fed the standard chow. In addition, total ambulation was also significantly lower in mice receiving the refined diet compared to standard chow fed mice (Fig. 2b). In line with these findings, IBA1 positive immunoreactive areas in histological slides of brain tissue, being a marker of microglia activation [34], were significantly larger in hippocampus (HIP) of refined diet- fed mice compared to standard chow-fed mice. Similar differences were not found in prefrontal cortex (PFC) (Fig. 2c–d).

### Effect of an extended intake of a refined diet on markers of intestinal barrier function and microbiota composition in C57BL/6 mice

**Fig. 3:**
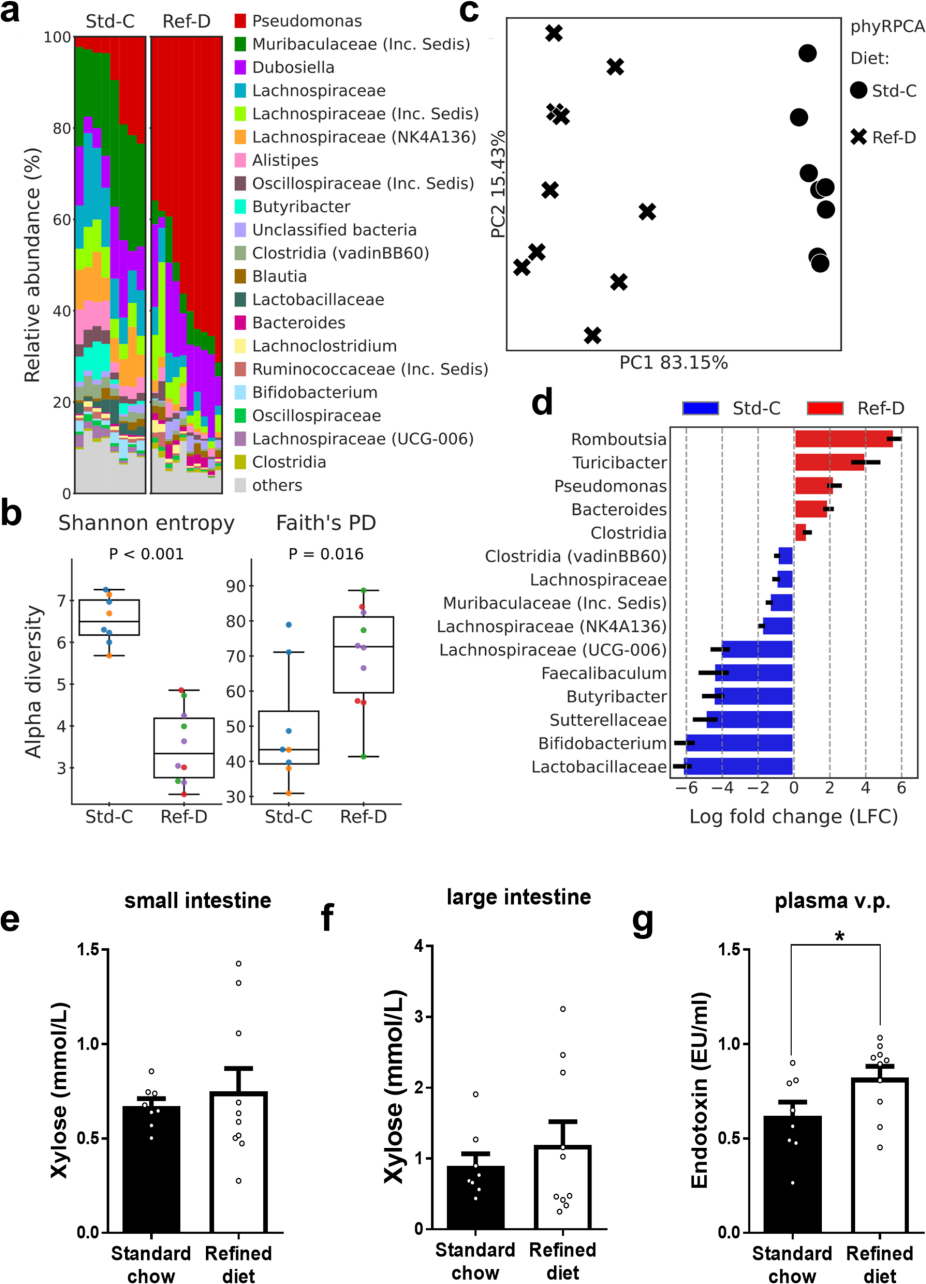
Effect of an extended intake of a refined diet or standard diet on markers of intestinal barrier and bacterial composition, diversity and abundances of bacteria in aging male C57BL/6 mice (**a**) Taxonomy area plots at the genus level (Silva 138.2). If genus level was not assigned, the last available taxonomy rank was used for the label. (**b**) Alpha diversity boxplots based on Shannon entropy and Faith’s phylogenetic diversity indices. Dots indicate individual samples and are colored by the cage. *P*-values of the ANOVA general test are plotted in the center of the upper part of each subplot. (**c**) Beta diversity PCoA plots based on phyRPCA. Marker shapes differentiate diets. (**d**) Differentially abundant (Ancom-BC) genera between Std-D (reference) and Ref-D (Red bars indicate taxa, significantly more abundant in Ref-D diet and blue for more abundant in Std-D). Xylose permeation of ex vivo everted gut sacs of (**e**) small intestine and (**f**) large intestine, (**g**) endotoxin concentration in plasma of aging mice receiving a standard chow (Std-C) or refined diet (Ref-D). Data are presented with means ± SEM. *p < 0.05. *n* = 8–10

As it has been suggested that the intake of a refined diet at least in part may add to the development of metabolic and neurological alterations through altering intestinal microbiota composition and barrier function [1–3, 35], we next determined intestinal microbiota composition by 16S rRNA gene sequencing. In the microbial communities (Fig. 3a) of mice fed standard chow, members of the Muribaculaceae, unclassified to the genus level, were dominant, followed by *Pseudomonas* and *Dubosiella*. In mice fed the refined diet a dominance was found in *Pseudomonas*, followed by *Dubosiella* and unclassified Muribaculaceae. The shift in bacterial communities was confirmed by the ANCOM-BC test (Fig. 3d) revealing that genera such as *Romboutsia*, *Turicibacter*, *Pseudomonas*, *Bacteroides* and unclassified Clostridia members were more abundant in mice fed with refined diet. At the same time, Clostridia being identified by the Silva database as vadinBB60, unclassified Lachnospiraceae (NK4A136, UCG-006), unclassified Muribaculaceae, Lactobacillaceae and Sutterellaceae, and genera *Faecalibaculum*, *Butyribacter* and *Bifidobacterium* had higher abundances in mice fed with the standard chow diet. Alpha diversity was also affected by the diet (Fig. 3b). Shannon entropy drastically decreased under the refined diet compared to the standard chow (*p* < 0.001). On the contrary, Faith’s phylogenetic diversity increased (*p* = 0.016). Samples from different dietary groups were also clustered separately on the PCoA plot based on phyRPCA distances (Fig. 3c). According to the Adonis test, after adjusting for the cages, diet was still a significant modifying factor (*p* = 0.001).

In contrast, neither in the small nor in the large intestine xylose permeation differed between feeding groups; however, data varied considerably (Fig. 3e–f). Bacterial endotoxin concentration in portal plasma was significantly higher in mice fed the refined diet when compared to those fed the standard chow (Fig. 3g).

### Effect of the addition of refined fibers, e.g., β-glucan isolated from oats and cellulose isolated from wood fiber to the refined diet on markers of senescence, glucose tolerance and liver as well as cognition

**Fig. 4:**
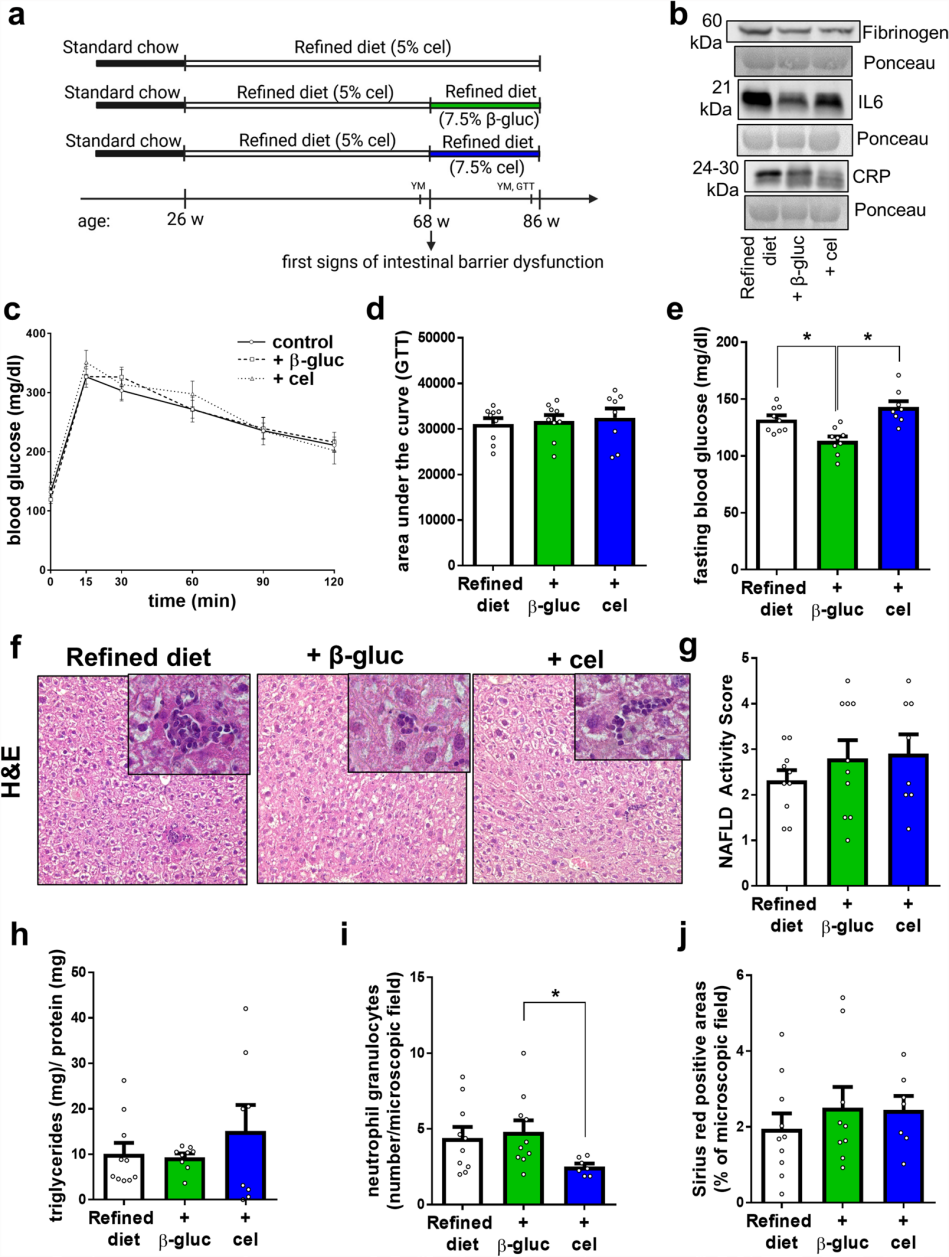
Effect of the addition of β-glucan isolated from oat and cellulose to the refined diet on glucose metabolism and liver damage in aging male C57BL/6 mice (**a**) Study design. (**b**) Representative blots of fibrinogen, interleukin 6 (IL6) and C-reactive protein (CRP) western blot bands in plasma. (**c**) Glucose concentration during glucose tolerance test (GTT) and (**d**) area under the curve of GTT, (**e**) fasting blood glucose, (**f**) representative pictures of hematoxylin and eosin stained liver (200 ×, 630 ×) and (**g**) evaluation via NAFLD activity score, (**h**) triglyceride concentration in liver homogenates, (**i**) number of neutrophil granulocytes and (**j**) Sirius red positive stained areas in liver sections in in mice receiving a refined diet or refined diet enriched with oat β-glucan or cellulose. w – weeks, YM – Y-Maze. Data are presented with means ± SEM. *p < 0.05.* n* = 7–10

Neither the exchange of cellulose and enrichment of the refined diet with β-glucan nor adding more cellulose to the refined diet (Study design see Fig. 4a) had any effect on markers of senescence or glucose levels during the GTT (Table 2, Fig. 4b–d). In contrast, fasting glucose levels were significantly lower in mice changed to the β-glucan enriched refined diet than in mice fed the refined diet or the cellulose enriched refined diet (Fig. 4e). Moreover, neither bodyweight and bodyweight gain during the intervention, nor NAFLD activity score, triglyceride levels, liver: body weight ratio and Sirius red stained areas differed between the three diet groups, while number of neutrophils was significantly lower in livers of mice fed the refined diet enriched with cellulose (Table 2, Fig. 4f–j).

**Table 2:** Effect of the addition of β-glucan isolated from oat and cellulose to the refined diet on body and liver weight and markers of senescence in aging male C57BL/6 mice

	Groups		
	Refined diet	+ β-glucan	+ cellulose
Absolute body weight (g)	35.9 ± 1.1	37.1 ± 0.8	37.7 ± 2.0
Absolute weight gain during intervention (g)	1.3 ± 0.4	0.8 ± 0.7	1.5 ± 0.7
Liver weight (g)	1.6 ± 0.1	1.6 ± 0.1	1.7 ± 0.1
Liver: body weight ratio (%)	4.6 ± 0.1	4.2 ± 0.1	4.5 ± 0.1
Fibrinogen/ponceau (% of refined diet)	100 ± 6.5	108 ± 20	89.7 ± 15
IL6/ponceau (% of refined diet)	100 ± 15	87.6 ± 10	91.1 ± 17
CRP/ponceau (% of refined diet)	100 ± 11	84.9 ± 8.1	78.7 ± 11

Data are presented with means ± SEM. CRP – C-reactive protein, IL6 – interleukin 6. *n* = 7–10

**Fig. 5:**
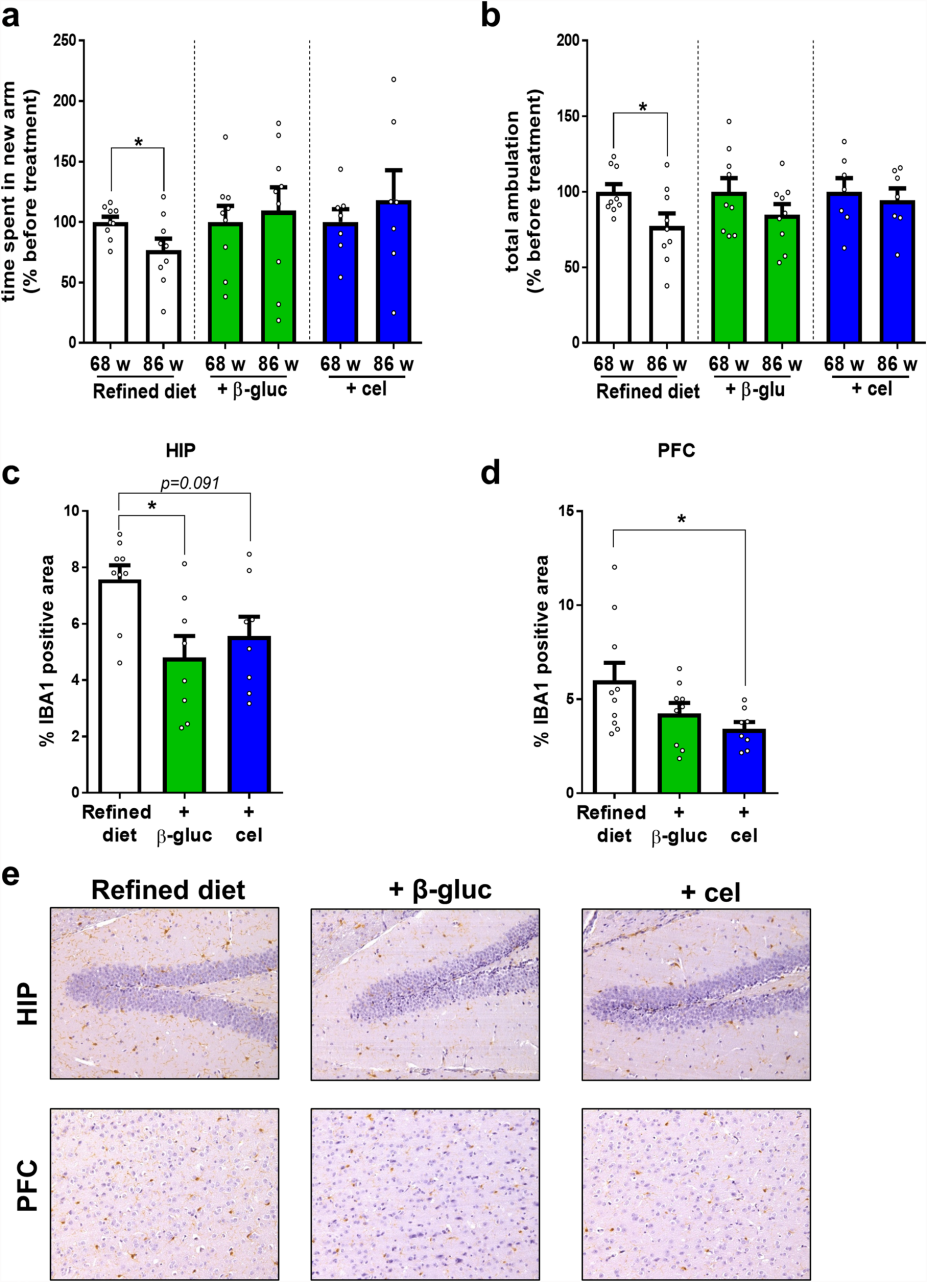
Effect of the addition of β-glucan isolated from oat and cellulose to the refined diet on cognitive alterations and markers of neuroinflammation in aging male C57BL/6 mice (**a**) Time spent in new arm and (**b**) total ambulation in Y-Maze, (**c**, **d**) ionized calcium-binding adapter molecule 1 (IBA1)-positive areas in hippocampus (HIP) and prefrontal cortex (PFC) and (**e**) representative pictures (200 ×) of aging mice receiving a refined diet or refined diet enriched with oat β-glucan or cellulose. Data are presented with means ± SEM. *p < 0.05. *n* = 7–10

Contrasting the findings for senescence and liver tissue, mice receiving either β-glucan or higher cellulose doses with their refined diet showed no decline in their cognition whereas both, time spent in new arm and total ambulation decreased significantly between 68 and 86 weeks in mice continuously fed the “normal” refined diet (Fig. 5a–b). Somewhat in line with these findings, the area of % IBA1 positive staining in HIP was significantly larger in mice continuously fed the refined diet compared to both other groups. In PFC, differences were only found when comparing measurements in PFC sections of mice fed the refined diet and those fed cellulose enriched diet (Fig. 4c–e).

### Effect of the addition of refined fibers, e.g., β-glucan isolated from oats and cellulose isolated from wood fiber to the refined diet on markers of intestinal permeability and intestinal microbiota

**Fig. 6:**
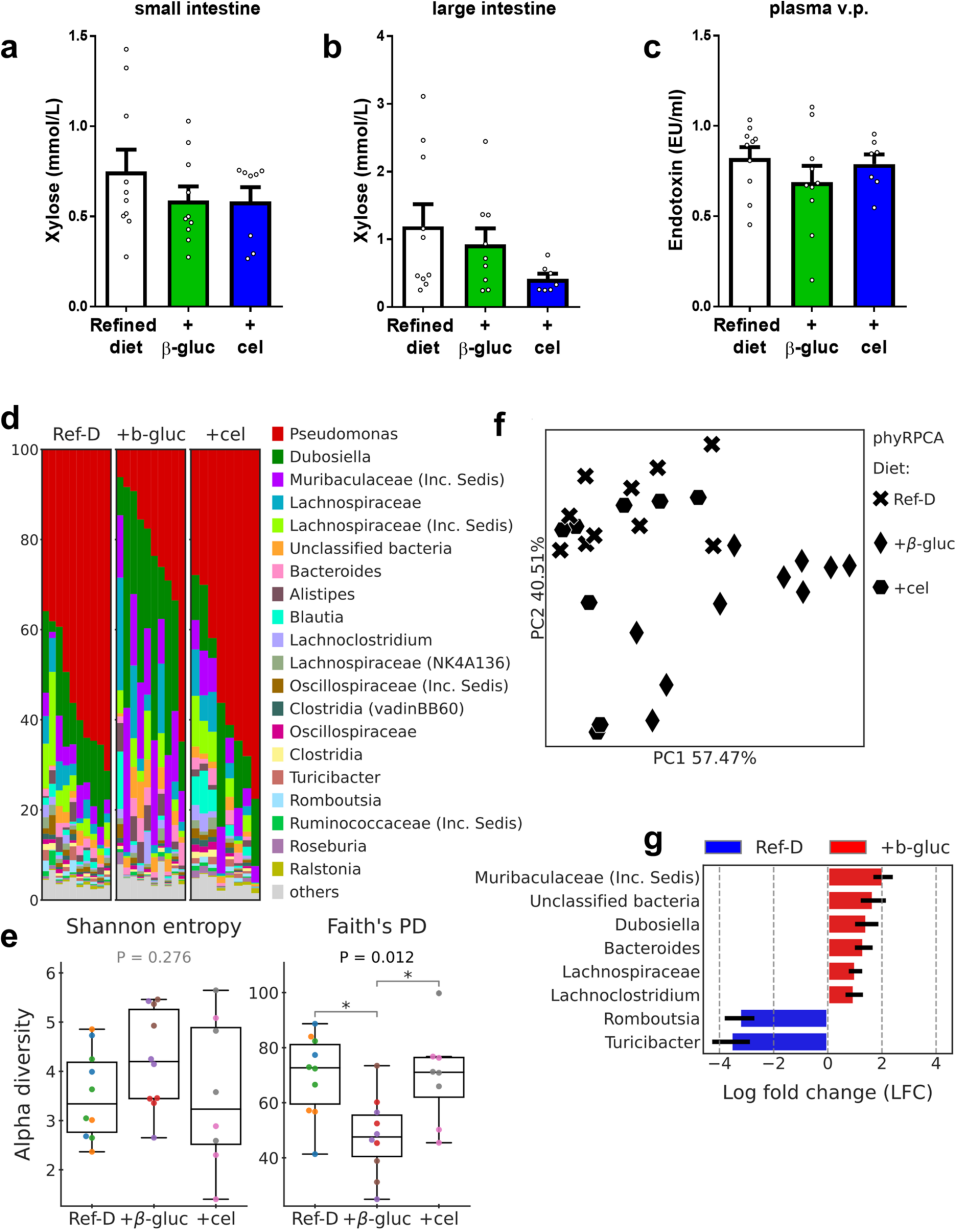
Effect of the addition of β-glucan isolated from oat and cellulose to the refined diet on intestinal barrier and bacterial composition, diversity and abundances in aging male C57BL/6 mice Xylose permeation of ex vivo everted gut sacs in (**a**) small and (**b**) large intestine and (**c**) endotoxin concentration in plasma. (**d**) Taxonomy area plots at the genus level (Silva 138.2). If genus level was not assigned, the last available taxonomy rank was used for the label. (**e**) Alpha diversity boxplots based on Shannon entropy and Faith’s phylogenetic diversity indices. Dots indicate individual samples and are colored by the cage. *P*-values of the ANOVA general test are plotted in the center of the upper part of each subplot. Adjusted *P*-values of the post hoc *t*-test are denoted by the asterisks for each pair (* = 0.05 > *P*-adjusted > 0.01). (**f**) Beta diversity PCoA plots based on phyRPCA. Marker shapes differentiate diets. (**g**) Differentially abundant (Ancom-BC) genera between Ref-D (reference) and diets with additions (Red bars indicate taxa, significantly more abundant in the + β-gluc diet and blue for more abundant in Ref-D) in in mice receiving a refined diet (Ref-D) or refined diet enriched with oat β-glucan (+ β-gluc) or cellulose (+ cel). Data are presented with means ± SEM. *p < 0.05. *n* = 7–10

Xylose permeation was similar between groups in both, small and large intestine. Moreover, bacterial endotoxin levels were also alike between feeding groups (Fig. 6a–c). The taxonomy profiles of mice fed the refined diet and the same diet with added cellulose were similar, as shown on the area plots (Fig. 6d) and confirmed by the ANCOM-BC test, which failed to detect differentially abundant genera. The addition of the β-glucan to the refined diet resulted in an increased abundance of unclassified Muribaculaceae and Lachnospiraceae, some unclassified bacteria, *Dubosiella, Bacteroides* and *Lachnoclostridium,* while the abundances of *Romboutsia* and *Turicibacter* decreased (Fig. 6g).

While no effect of the addition of either dietary fiber to the refined diet was detected when testing Shannon entropy, Faith’s phylogenetic diversity was significantly affected (*P* = 0.012). Pairwise comparisons revealed that the addition of β-glucan to the diet decreased Faith’s phylogenetic diversity compared to the other two feeding groups (refined diet: *P-adj* = 0.021 and cellulose-enriched diet: *P-adj* = 0.019) (Fig. 6e). Beta diversity, as expressed by phyRPCA distances, was also affected by the supplementation (*P* = 0.001). β-glucan supplemented samples differed from those of the refined diet (*P-adj* = 0.003) and the diet fortified with cellulose (*P-adj* = 0.003) (Fig. 6f).

## Discussion

A diet rich in highly processed foods is discussed to be critical in the development of overweight and several non-communicable diseases [36, 37]. Data on the long-term effect of the intake of a highly refined diet with respect to (healthy) aging are limited. In the present study, old-aged mice fed ad libitum a refined diet lacking natural ingredients found in “standard” chow thereby somewhat mimicking highly processed foods in human diet, were compared to mice fed standard chow. Body weight gain was similar between groups. In contrast, signs of aging-related decline including markers of senescence in blood, glucose tolerance, and parameters of liver health like inflammation and fat accumulation were markedly more progressed in mice fed the refined diet when compared to age-matched mice fed the standard chow. Moreover, cognitive decline was also more progressed in mice fed the refined diet. These findings are somewhat in line with those of others feeding a refined diet to young mice [38]. Indeed, it has been shown before in young mice that the intake of a low-fat purified diet was related with hepatic fat accumulation and inflammatory alterations in the liver which were not found when mice were fed a standard, grain-based chow. Moreover, markers of glucose homeostasis were also found to be altered in young mice fed a low-fat purified diet [38]. Taken together, results of the present study further bolster the hypothesis that the intake of a refined diet enhances the aging process in mice being related with marked metabolic and cognitive alterations. Our results by no means preclude that alterations alike also develop in mice fed standard chow; however, the development may take longer.

### The enhanced onset of aging related alterations in mice consuming a refined diet is related with alterations of intestinal microbiota composition and elevated bacterial endotoxin levels in portal blood

Results of several studies suggest that both, changes of intestinal microbiota composition and impairments of intestinal barrier function subsequently leading to an increased translocation of bacterial endotoxin may be critical in the development of aging related decline and herein especially inflammatory process in the liver but also the central nervous system [9–12]. Indeed, it has been shown by us and others before that even healthy aging is related with a marked shift in intestinal microbiota composition [11, 12, 39, 40]. Moreover, it has been shown that bacterial endotoxin levels in portal and peripheral blood gradually increase with age being paralleled with impairments of intestinal barrier function especially in small intestinal tissue [10–12]. In the present study, intestinal microbiota composition in the colon differed markedly between old-aged mice fed standard chow and those fed the refined diet with the latter showing lower alpha diversity as assessed by Shannon entropy, but higher Faith PD. Such a pattern indicates that despite the microbiota entropy of mice fed with a refined diet becoming lower, the phylogenetic diversity between remaining bacteria increased. Studies in young mice fed a purified diet have shown not only alterations in liver health and glucose metabolism as well as cognitive health but also, that this was linked to a marked change in intestinal microbiota composition [3, 38]. Specifically, diversity indices used in these studies revealed a marked decrease in bacterial richness and evenness in mice fed the purified diet [38]. It has been suggested before that cognitive impairments in aging humans and mice are related to a lower alpha diversity [10, 41]. Moreover, there was a higher abundance of *Pseudomonas* and *Dubosiella* in mice fed the refined diet. Both have been discussed to be related to the development of cognitive impairments but also liver diseases like metabolic dysfunction-associated steatotic liver disease (MASLD) [42–44]. However, contrasting the findings in the present study, results of others suggest that a reduction of *Dubosiella* has been related to the development of aging related cognitive impairments and steatotic liver [43, 45, 46] while a higher prevalence of the *Dubosiella* has been shown to impede Alzheimer´s disease and MASLD probably through an enhanced synthesis of palmitoleic acids [44]. *Pseudomonas* genus includes species that are opportunistic pathogens, and in a healthy gut, their abundance is typically low [47]. A relative dominance of *Pseudomonas* could be an indication of a gut dysbiosis and potentially reflect a pro-inflammatory or metabolically altered gut environment. Further studies are needed to clarify this but also the impact of different bacterial genera and, even more so, strains in aging related decline and especially the development of cognitive impairments and liver decline.

While no differences were found between old-aged mice fed standard chow and those fed the refined diet with respect to xylose permeation, bacterial endotoxin levels were higher in the latter. The apparent contrast in findings for bacterial endotoxin levels and intestinal permeability measurements might have been related to the use of xylose as markers. Indeed, the molecular mass of xylose but also other sugars often employed in intestinal permeability measurements is smaller than that of bacterial endotoxin. Aggregates of bacterial endotoxin sizes up to 1,000 kDA, while sugars, such as xylose are markedly smaller with respect to molecular size (0.15 kDa) [48–50]. Therefore, it could be that while intestinal barrier dysfunction was more pronounced in mice fed the refined diet xylose permeation was still similar between groups. However, it could also be that bacterial turnover was higher, and subsequently more bacterial endotoxin was released in mice fed the refined diet. This needs to be determined in future studies. In addition, we have previously shown that healthy aging in mice is associated with impairments in the small intestinal barrier, while in these studies the colonic barrier seems to be rather unaffected [10, 11]. Still, in line with previous studies the higher bacterial endotoxin levels found in the present study were related to a more pronounced NAFLD activity score and inflammatory alterations in liver tissue as well as the central nervous system [9, 12, 51, 52].

In summary, results of our study suggest that while not affecting intestinal barrier function directly, intestinal microbiota composition was markedly altered in mice consuming the refined diet being related to higher bacterial endotoxin levels in portal blood. However, further studies are needed to clarify the interaction of diet and bacterial strains and their subsequent effect on metabolism and cognition in the development of aging related decline.

### Fortifying a refined diet with soluble or insoluble fiber has no effect on aging-related liver decline and changes in glucose tolerance but diminished the onset of cognitive decline and inflammation in PFC and HIP

Results of an umbrella review suggest that an adherence to a healthy dietary pattern is related to better cognitive performance in older age [53]. In line with these findings, diet has also been suggested to be one of the key triggers of liver diseases [54, 55] and that the development of MASLD is frequently related to lower total fiber intake [56–58]. Somewhat contrasting these findings, results of meta-analysis suggest that supplementing human diet with pre- and probiotics as well as fermented food, respectively, has limited effect regarding cognitive outcomes [55] while development of metabolic liver diseases has been shown to be alleviated when diet was enriched with fibers [57, 58]. In the present study the data suggest that the addition of refined fibers to the refined diet, regardless if soluble or insoluble, had limited effects with respect to the onset of senescence and related metabolic alterations e.g., liver damage and glucose metabolism. In contrast, our data suggest that the addition of fiber to the diet dampened cognitive impairments and inflammatory alterations in the central nervous system. Interestingly, the lessening of impairments of cognitive function was related to changes in beta-diversity of intestinal microbiota while not affecting bacterial endotoxin levels in portal blood. Also, in mice receiving the diet enriched with oat-derived β-glucan, a higher relative abundance of unclassified *Muribaculaceae* and *Lachnospiraceae*, some unclassified bacteria, *Dubosiella*, *Bacteroides* and *Lachnoclostridium* was detected, while the relative abundances of *Romboutsia* and *Turicibacter* was lower. All of these bacteria have been suggested to have biological importance in gut health. For example, *Muribaculaceae* bacteria have been shown to be often fiber-degrading and to produce short-chain fatty acids (SCFAs) like propionate and butyrate [59]. The latter have been shown to have anti-inflammatory properties and support gut barrier integrity as well as the energy metabolism of colonocytes but also affect the development of MASLD [60–62]. Their enrichment could suggest improved microbial fermentation of fiber. Moreover, *Lachnospiraceae* are also known for butyrate production [63]. Also, a lower relative abundance of *Romboutsia* has been associated with high-fiber diets [64]. It could be that the lower relative abundance of this bacterial strain may signal a shift away from an inflammatory or dysbiotic profile. Some bacteria of the *Turicibacter* genus have been shown to correlate with alterations in dietary fat and body weight and may influence host metabolites like lipids and bile acids [65]. Further studies are needed especially in old aged rodents and humans to clarify if these shifts in the relative abundance are reproducible upon a change in diet and their impact on the aging process. Still, the data suggest that the composition of intestinal microbiota and derived metabolites may be drivers of aging related cognitive decline while bacterial endotoxin may be more critical with respect to liver damage and metabolic alterations. Indeed, studies of others suggest that an association of microbiota and especially aromatic amino acids metabolism is associated with short-term and working memory [66]. It also has been suggested that a fortification of the diet with fibers may increase the bacteria mediated formation of SCFA such as butyrate [67] and that in older rodents the prevalence of butyrate-producing bacteria is lower than in young mice [68]. Moreover, in the same study, it has been shown that in old mice a supplementation of butyrate may improve cognitive function and decrease inflammatory alterations in brain tissue being also related to an improved intestinal barrier function [68]. In line with this, others also reported that an increased intake of fiber was related to an increased formation of SCFA and beneficial effect on cognitive function. However, studies showing protective effects of SCFA supplementation on cognitive function also reported protective effects on intestinal barrier function [68]. Therefore, it could be that in the present study, the protective effects of either of the fibers supplemented were related to other mechanisms than an enhanced formation of SCFA. However, these will have to be determined in future studies.

In the present study, neither markers of intestinal barrier function e.g., xylose permeation and bacterial endotoxin levels in blood nor liver parameters were altered in mice concomitantly fed either of the fibers. Results of our own studies suggest that aging related cognitive decline and inflammatory alterations in the central nervous system are also related to increased bacterial endotoxin levels [9, 10, 12]. In contrast to our findings, previous studies of others reported positive effects of dietary fiber supplementation on intestinal barrier dysfunction in aging mice, as well as in mice with diet-induced metabolic diseases [69, 70]. The differences between these studies and the present study may have resulted from differences in dosing and duration but also age of mice. It has also been suggested that soluble and insoluble dietary fiber do not always exert similar effects on intestinal barrier function [71]. 

This study has some limitations which need to be taken into consideration when interpreting the data. First, the experiments were only performed in male mice limiting the generalization to other sex and species. While mice are one of the most widely used animal model for aging-associated experiments [72] and several metabolic pathways and alterations related to aging seem similar between mice and humans, there are differences including a different intestinal microbiome and higher metabolic rate [73], which may also affect the effects of dietary fiber on health. Moreover, while our study suggests that the addition of fiber to the refined diet had limited effects on markers of intestinal barrier function and intestinal microbiota as well as liver tissue, further studies are needed to study intestinal barrier and microbiota as well as liver metabolism in more depth. Indeed, we found some shifts in the relative abundance of bacterial species that have been shown to be able to produce SCFA. Therefore, it could be that some of the beneficial effects found may have resulted from an increased synthesis of SCFA. Due to a lack of suitable samples, the latter were not assessed in the present study. Also, while in the present study some effects on cognition were found, additional studies focusing more on different areas of memory and different brain regions are needed to determine 1) if effects are only found in specific areas of the brain in mice and 2) if similar effects are also found in elderly humans. Also, intestinal permeability was only assessed by xylose permeation in ex vivo everted gut sac experiments. The evaluation of other direct markers of intestinal barrier function, such as FITC-dextran in vivo [74] will need to confirm the role of intestinal barrier function in refined diet accelerated aging.

## Conclusion

In summary, results of our study further bolster the hypothesis that highly processed and refined foods may affect health and thereby also healthy aging. Indeed, our results suggest that in male mice a long-term intake of a refined diet may accelerate the onset of senescence being related with inflammatory alterations in liver, impairments of glucose metabolism and cognition. Also, our results suggest that the enhanced onset of aging related decline in male mice is related to differences in intestinal microbiota composition and higher bacterial endotoxin levels in portal blood. Moreover, our results also suggest that fortifying a refined diet with isolated refined fibers -be they soluble or insoluble- has only limited to no effect on metabolic alterations but may alleviate the enhanced onset of cognitive impairments and inflammatory alterations in brain tissue of rodents. Further studies are needed to determine molecular mechanisms underlying the interaction of especially refined diets and added fibers with intestinal microbiota and permeation of bacterial endotoxin and the aging process in mice. Moreover, it remains to be determined how and if the intake of highly or ultra-processed foods affect aging in humans and if an addition of fiber to the (highly/ultra-processed) diet exerts effects alike in humans.

## Supplementary Information

Below is the link to the electronic supplementary material.

Supplementary file1 (PDF 54.4 KB)

## Data Availability

Data will be made available on reasonable request. Raw sequences were deposited to the European Nucleotide Archive (ENA) under accession number PRJEB80087.

## Abbreviations

BSA: bovine serum albumin
CRP: C-reactive protein
Faith’s PD: Faith’s phylogenetic diversity
GTT: Glucose tolerance test
HIP: hippocampus
IBA1: ionized calcium-binding adapter molecule 1
IL6: interleukin 6
KRH buffer: 1x Krebs-Henseleit-bicarbonate buffer
MASLD: metabolic dysfunction-associated steatotic liver disease
PFC: prefrontal cortex
SEM: standard error of means
SCFA: short chain fatty acids

## References

[1] Rauber F, Louzada MLdC, Chang K, Huybrechts I, Gunter MJ, Monteiro CA, et al. Implications of food ultra-processing on cardiovascular risk considering plant origin foods: an analysis of the UK biobank cohort. Lancet Reg Health. 2024;43:100948. 10.1016/j.lanepe.2024.100948. 10.1016/j.lanepe.2024.100948 PMC11360147 39210945

[2] Barbaresko J, Broder J, Conrad J, Szczerba E, Lang A, Schlesinger S. Ultra-processed food consumption and human health: an umbrella review of systematic reviews with meta-analyses. Crit Rev Food Sci Nutr. 2024. 10.1080/10408398.2024.2317877. 38363072 10.1080/10408398.2024.2317877

[3] Connell E, Blokker B, Kellingray L, Le Gall G, Philo M, Pontifex MG, et al. Refined diet consumption increases neuroinflammatory signalling through bile acid dysmetabolism. Nutr Neurosci. 2024;27(10):1088–101. 10.1080/1028415X.2023.2301165. 38170169 10.1080/1028415X.2023.2301165

[4] Batdorf HM, Lawes LL, Cassagne GA, Fontenot MS, Harvey IC, Richardson JT, et al. Accelerated onset of diabetes in non-obese diabetic mice fed a refined high-fat diet. Diabetes Obes Metab. 2024;26(6):2158–66. 10.1111/dom.15522. 38433703 10.1111/dom.15522 PMC11078605

[5] Schipke J, Brandenberger C, Vital M, Muhlfeld C. Starch and fiber contents of purified control diets differentially affect hepatic lipid homeostasis and gut microbiota composition. Front Nutr. 2022;9:915082. 10.3389/fnut.2022.915082. 35873446 10.3389/fnut.2022.915082 PMC9301012

[6] Weiskirchen S, Weiper K, Tolba RH, Weiskirchen R. All you can feed: some comments on production of mouse diets used in biomedical research with special emphasis on non-alcoholic fatty liver disease research. Nutrients. 2020;12(1):163. 10.3390/nu12010163. 31936026 10.3390/nu12010163 PMC7019265

[7] Sanchez-Morate E, Gimeno-Mallench L, Stromsnes K, Sanz-Ros J, Roman-Dominguez A, Parejo-Pedrajas S, et al. Relationship between diet, microbiota, and healthy aging. Biomedicines. 2020. 10.3390/biomedicines8080287. 32823858 10.3390/biomedicines8080287 PMC7460310

[8] Wu S, Liu X, Jiang R, Yan X, Ling Z. Roles and mechanisms of gut microbiota in patients with Alzheimer’s disease. Front Aging Neurosci. 2021;13:650047. 10.3389/fnagi.2021.650047. 34122039 10.3389/fnagi.2021.650047 PMC8193064

[9] Jin CJ, Baumann A, Brandt A, Engstler AJ, Nier A, Hege M, et al. Aging-related liver degeneration is associated with increased bacterial endotoxin and lipopolysaccharide binding protein levels. Am J Physiol Gastrointest Liver Physiol. 2020;318(4):G736–47. 10.1152/ajpgi.00345.2018. 32090603 10.1152/ajpgi.00345.2018

[10] Brandt A, Kromm F, Hernandez-Arriaga A, Martinez Sanchez I, Bozkir HO, Staltner R, et al. Cognitive alterations in old mice are associated with intestinal barrier dysfunction and induced Toll-like receptor 2 and 4 signaling in different brain regions. Cells. 2023. 10.3390/cells12172153. 37681885 10.3390/cells12172153 PMC10486476

[11] Brandt A, Baumann A, Hernandez-Arriaga A, Jung F, Nier A, Staltner R, et al. Impairments of intestinal arginine and NO metabolisms trigger aging-associated intestinal barrier dysfunction and “inflammaging.” Redox Biol. 2022;58:102528. 10.1016/j.redox.2022.102528. 36356464 10.1016/j.redox.2022.102528 PMC9649383

[12] Baumann A, Hernandez-Arriaga A, Brandt A, Sanchez V, Nier A, Jung F, et al. Microbiota profiling in aging-associated inflammation and liver degeneration. Int J Med Microbiol. 2021;311(4):151500. 10.1016/j.ijmm.2021.151500. 33813306 10.1016/j.ijmm.2021.151500

[13] Kuhn F, Adiliaghdam F, Cavallaro PM, Hamarneh SR, Tsurumi A, Hoda RS, et al. Intestinal alkaline phosphatase targets the gut barrier to prevent aging. JCI Insight. 2020. 10.1172/jci.insight.134049. 32213701 10.1172/jci.insight.134049 PMC7213802

[14] Ding S, Cheng Y, Azad MAK, Dong H, He J, Huang P, et al. Dietary fiber alters immunity and intestinal barrier function of different breeds of growing pigs. Front Immunol. 2023;14:1104837. 10.3389/fimmu.2023.1104837. 36865532 10.3389/fimmu.2023.1104837 PMC9972983

[15] Erlanson-Albertsson C, Stenkula KG. The importance of food for endotoxemia and an inflammatory response. Int J Mol Sci. 2021. 10.3390/ijms22179562. 34502470 10.3390/ijms22179562 PMC8431640

[16] Zhang Y, Zhu X, Yu X, Novak P, Gui Q, Yin K. Enhancing intestinal barrier efficiency: A novel metabolic diseases therapy. Front Nutr. 2023;10:1120168. 10.3389/fnut.2023.1120168. 36937361 10.3389/fnut.2023.1120168 PMC10018175

[17] McRorie JW Jr., McKeown NM. Understanding the physics of functional fibers in the gastrointestinal tract: an evidence-based approach to resolving enduring misconceptions about insoluble and soluble fiber. J Acad Nutr Diet. 2017;117(2):251–64. 10.1016/j.jand.2016.09.021. 27863994 10.1016/j.jand.2016.09.021

[18] Mathews R, Kamil A, Chu Y. Global review of heart health claims for oat beta-glucan products. Nutr Rev. 2020;78(Supplement_1):78–97. 10.1093/nutrit/nuz069. 32728751 10.1093/nutrit/nuz069

[19] Opperman C, Majzoobi M, Farahnaky A, Shah R, Van TTH, Ratanpaul V, et al. Beyond soluble and insoluble: a comprehensive framework for classifying dietary fibre’s health effects. Food Res Int. 2025;206:115843. 10.1016/j.foodres.2025.115843. 40058888 10.1016/j.foodres.2025.115843

[20] Fischer F, Romero R, Hellhund A, Linne U, Bertrams W, Pinkenburg O, et al. Dietary cellulose induces anti-inflammatory immunity and transcriptional programs via maturation of the intestinal microbiota. Gut Microbes. 2020;12(1):1–17. 10.1080/19490976.2020.1829962. 33079623 10.1080/19490976.2020.1829962 PMC7583510

[21] Kleiner DE, Brunt EM, Van Natta M, Behling C, Contos MJ, Cummings OW, Ferrell LD, Liu YC, Torbenson MS, Unalp-Arida A, Yeh M, McCullough AJ, Sanyal AJ, N. Nonalcoholic Steatohepatitis Clinical Research, Design and validation of a histological scoring system for nonalcoholic fatty liver disease. Hepatology, 2005. 41(6): 1313–21. 10.1002/hep.20701. 10.1002/hep.20701 15915461

[22] Spruss A, Kanuri G, Stahl C, Bischoff SC, Bergheim I. Metformin protects against the development of fructose-induced steatosis in mice: role of the intestinal barrier function. Lab Invest. 2012;92(7):1020–32. 10.1038/labinvest.2012.75. 22525431 10.1038/labinvest.2012.75

[23] Rajcic D, Baumann A, Hernandez-Arriaga A, Brandt A, Nier A, Jin CJ, et al. Citrulline supplementation attenuates the development of non-alcoholic steatohepatitis in female mice through mechanisms involving intestinal arginase. Redox Biol. 2021;41:101879. 10.1016/j.redox.2021.101879. 33550112 10.1016/j.redox.2021.101879 PMC7868995

[24] Sanchez V, Baumann A, Kromm F, Yergaliyev T, Brandt A, Scholda J, et al. Oral supplementation of choline attenuates the development of alcohol-related liver disease (ALD). Mol Med. 2024;30(1):181. 10.1186/s10020-024-00950-4. 39425011 10.1186/s10020-024-00950-4 PMC11488139

[25] Hernandez-Arriaga A, Baumann A, Witte OW, Frahm C, Bergheim I, Camarinha-Silva A. Changes in oral microbial ecology of C57BL/6 mice at different ages associated with sampling methodology. Microorganisms. 2019. 10.3390/microorganisms7090283. 31443509 10.3390/microorganisms7090283 PMC6780121

[26] Bolyen E, Rideout JR, Dillon MR, Bokulich NA, Abnet CC, Al-Ghalith GA, et al. Reproducible, interactive, scalable and extensible microbiome data science using QIIME 2. Nat Biotechnol. 2019;37(8):852–7. 10.1038/s41587-019-0209-9. 31341288 10.1038/s41587-019-0209-9 PMC7015180

[27] Martin M. Cutadapt removes adapter sequences from high-throughput sequencing reads. EMBnet j. 2011;17(1):10–2.

[28] Callahan BJ, McMurdie PJ, Rosen MJ, Han AW, Johnson AJ, Holmes SP. DADA2: High-resolution sample inference from Illumina amplicon data. Nat Methods. 2016;13(7):581–3. 10.1038/nmeth.3869. 27214047 10.1038/nmeth.3869 PMC4927377

[29] Rognes T, Flouri T, Nichols B, Quince C, Mahé F. VSEARCH: a versatile open source tool for metagenomics. PeerJ. 2016;4:e2584. 27781170 10.7717/peerj.2584 PMC5075697

[30] Pedregosa F, G. Varoquau G, Gramfort A, Michel V, Thirion B, Grisel O, Blondel M, Prettenhofer P, Weiss R, Dubourg V. Scikit-learn: Machine learning in Python. J Mach Learn Res. 2011;12:2825–2830.

[31] Quast C, Pruesse E, Yilmaz P, Gerken J, Schweer T, Yarza P, Peplies J, Glöckner FO. The SILVA ribosomal RNA gene database project: improved data processing and web-based tools. Nucleic Acids Res 2013;41(Database issue): D590–6. 10.1093/nar/gks1219. 10.1093/nar/gks1219 PMC3531112 23193283

[32] Robeson MS, O’Rourke DR, Kaehler BD, Ziemski M, Dillon MR, Foster JT, Bokulich NA. RESCRIPt: Reproducible sequence taxonomy reference database management. PLoS Comput Biol. 2021;17(11):e1009581. 10.1371/journal.pcbi.1009581. 10.1371/journal.pcbi.1009581 PMC8601625 34748542

[33] Martino C, Morton JT, Marotz CA, Thompson LR, Tripathi A, Knight R, et al. A novel sparse compositional technique reveals microbial perturbations. mSystems. 2019. 10.1128/mSystems.00016-19. 30801021 10.1128/mSystems.00016-19 PMC6372836

[34] Sasaki Y, Ohsawa K, Kanazawa H, Kohsaka S, Imai Y. Iba1 is an actin-cross-linking protein in macrophages/microglia. Biochem Biophys Res Commun. 2001;286(2):292–7. 10.1006/bbrc.2001.5388. 11500035 10.1006/bbrc.2001.5388

[35] Iversen KN, Dicksved J, Zoki C, Fristedt R, Pelve EA, Langton M, et al. The effects of high fiber rye, compared to refined wheat, on gut microbiota composition, plasma short chain fatty acids, and implications for weight loss and metabolic risk factors (the Ryeweight study). Nutrients. 2022. 10.3390/nu14081669. 35458231 10.3390/nu14081669 PMC9032876

[36] Aramburu A, Alvarado-Gamarra G, Cornejo R, Curi-Quinto K, Diaz-Parra CDP, Rojas-Limache G, et al. Ultra-processed foods consumption and health-related outcomes: a systematic review of randomized controlled trials. Front Nutr. 2024;11:1421728. 10.3389/fnut.2024.1421728. 38988861 10.3389/fnut.2024.1421728 PMC11233771

[37] Chen X, Zhang Z, Yang H, Qiu P, Wang H, Wang F, et al. Consumption of ultra-processed foods and health outcomes: a systematic review of epidemiological studies. Nutr J. 2020;19(1):86. 10.1186/s12937-020-00604-1. 32819372 10.1186/s12937-020-00604-1 PMC7441617

[38] Daniel N, Rossi Perazza L, Varin TV, Trottier J, Marcotte B, St-Pierre P, et al. Dietary fat and low fiber in purified diets differently impact the gut-liver axis to promote obesity-linked metabolic impairments. Am J Physiol Gastrointest Liver Physiol. 2021;320(6):G1014–33. 10.1152/ajpgi.00028.2021. 33881354 10.1152/ajpgi.00028.2021

[39] Langille MG, Meehan CJ, Koenig JE, Dhanani AS, Rose RA, Howlett SE, et al. Microbial shifts in the aging mouse gut. Microbiome. 2014;2(1):50. 10.1186/s40168-014-0050-9. 25520805 10.1186/s40168-014-0050-9 PMC4269096

[40] Wu ML, Yang XQ, Xue L, Duan W, Du JR. Age-related cognitive decline is associated with microbiota-gut-brain axis disorders and neuroinflammation in mice. Behav Brain Res. 2021;402:113125. 10.1016/j.bbr.2021.113125. 33422597 10.1016/j.bbr.2021.113125

[41] Verdi S, Jackson MA, Beaumont M, Bowyer RCE, Bell JT, Spector TD, et al. An investigation into physical frailty as a link between the gut microbiome and cognitive health. Front Aging Neurosci. 2018;10:398. 10.3389/fnagi.2018.00398. 30564113 10.3389/fnagi.2018.00398 PMC6288358

[42] Rashid MI, Rashid H, Andleeb S, Ali A. Evaluation of Blood-Brain-Barrier Permeability, Neurotoxicity, and Potential Cognitive Impairment by Pseudomonas aeruginosa’s Virulence Factor Pyocyanin. Oxid Med Cell Longev. 2022;2022:3060579. 10.1155/2022/3060579. 35340215 10.1155/2022/3060579 PMC8948603

[43] Gao S, He Y, Zhang L, Liu L, Qu C, Zheng Z, Miao J. Conjugated linoleic acid ameliorates hepatic steatosis by modulating intestinal permeability and gut microbiota in ob/ob mice. Food Nutr Res, 2022:66. 10.29219/fnr.v66.8226. 10.29219/fnr.v66.8226 PMC8941409 35382379

[44] Chen Y, Li Y, Fan Y, Chen S, Chen L, Chen Y, et al. Gut microbiota-driven metabolic alterations reveal gut-brain communication in Alzheimer’s disease model mice. Gut Microbes. 2024;16(1):2302310. 10.1080/19490976.2024.2302310. 38261437 10.1080/19490976.2024.2302310 PMC10807476

[45] Jing Y, Wang Q, Bai F, Li Z, Li Y, Liu W, et al. Role of microbiota-gut-brain axis in natural aging-related alterations in behavior. Front Neurosci. 2024;18:1362239. 10.3389/fnins.2024.1362239. 38699678 10.3389/fnins.2024.1362239 PMC11063250

[46] Ye X, Sun P, Lao S, Wen M, Zheng R, Lin Y, et al. Fgf21-Dubosiella axis mediates the protective effects of exercise against NAFLD development. Life Sci. 2023;334:122231. 10.1016/j.lfs.2023.122231. 37935276 10.1016/j.lfs.2023.122231

[47] Diggle SP, Whiteley M. Microbe profile: Pseudomonas aeruginosa: opportunistic pathogen and lab rat. Microbiology (Reading). 2020;166(1):30–3. 10.1099/mic.0.000860. 31597590 10.1099/mic.0.000860 PMC7273324

[48] Demmer W, Fischer-Frühholz S, Nußbaumer D, Melzner D. Chapter 14 - Economic production of biopharmaceuticals by high-speed membrane adsorbers. In Membrane Science and Technology, Bhattacharyya D. and Butterfield DA, Editors. 2003;283–295, Elsevier.

[49] Rietschel ET, Kirikae T, Schade FU, Mamat U, Schmidt G, Loppnow H, et al. Bacterial endotoxin: molecular relationships of structure to activity and function. FASEB J. 1994;8(2):217–25. 10.1096/fasebj.8.2.8119492. 8119492 10.1096/fasebj.8.2.8119492

[50] Vojdani A. For the assessment of intestinal permeability, size matters. Altern Ther Health Med. 2013;19(1):12–24. 23341423

[51] Soppert J, Brandt EF, Heussen NM, Barzakova E, Blank LM, Kuepfer L, et al. Blood endotoxin levels as biomarker of nonalcoholic fatty liver disease: a systematic review and meta-analysis. Clin Gastroenterol Hepatol. 2023;21(11):2746–58. 10.1016/j.cgh.2022.11.030. 36470528 10.1016/j.cgh.2022.11.030

[52] Chmielarz M, Sobieszczanska B, Sroda-Pomianek K. Metabolic endotoxemia: from the Gut to Neurodegeneration. Int J Mol Sci. 2024;25(13). 10.3390/ijms25137006. 10.3390/ijms25137006 PMC11241432 39000116

[53] Khoshdooz S, Bonyad A, Bonyad R, Khoshdooz P, Jafari A, Rahnemayan S, et al. Role of dietary patterns in older adults with cognitive disorders: an umbrella review utilizing neuroimaging biomarkers. Neuroimage. 2024;303:120935. 10.1016/j.neuroimage.2024.120935. 39547460 10.1016/j.neuroimage.2024.120935

[54] Gao V, Long MT, Singh SR, Kim Y, Zhang X, Rogers G, et al. A healthy diet is associated with a lower risk of hepatic fibrosis. J Nutr. 2023;153(5):1587–96. 10.1016/j.tjnut.2023.03.038. 37023964 10.1016/j.tjnut.2023.03.038 PMC10273161

[55] Marx W, Scholey A, Firth J, D’Cunha NM, Lane M, Hockey M, et al. Prebiotics, probiotics, fermented foods and cognitive outcomes: A meta-analysis of randomized controlled trials. Neurosci Biobehav Rev. 2020;118:472–84. 10.1016/j.neubiorev.2020.07.036. 32860802 10.1016/j.neubiorev.2020.07.036

[56] Nier A, Huber Y, Labenz C, Michel M, Bergheim I, Schattenberg JM. Adipokines and endotoxemia correlate with hepatic steatosis in non-alcoholic fatty liver disease (NAFLD). Nutrients. 2020. 10.3390/nu12030699. 32151020 10.3390/nu12030699 PMC7146245

[57] Zhu Y, Yang H, Zhang Y, Rao S, Mo Y, Zhang H, et al. Dietary fiber intake and non-alcoholic fatty liver disease: The mediating role of obesity. Front Public Health. 2022;10:1038435. 10.3389/fpubh.2022.1038435. 36684870 10.3389/fpubh.2022.1038435 PMC9853063

[58] Stachowska E, Portincasa P, Jamiol-Milc D, Maciejewska-Markiewicz D, Skonieczna-Zydecka K. The relationship between prebiotic supplementation and anthropometric and biochemical parameters in patients with NAFLD-A systematic review and meta-analysis of randomized controlled trials. Nutrients. 2020;12(11). 10.3390/nu12113460. 10.3390/nu12113460 PMC7698299 33187278

[59] Zhu Y, Chen B, Zhang X, Akbar MT, Wu T, Zhang Y, et al. Exploration of the Muribaculaceae family in the gut microbiota: diversity, metabolism, and function. Nutrients. 2024. 10.3390/nu16162660. 39203797 10.3390/nu16162660 PMC11356848

[60] Baumann A, Jin CJ, Brandt A, Sellmann C, Nier A, Burkard M, et al. Oral supplementation of sodium butyrate attenuates the progression of non-alcoholic steatohepatitis. Nutrients. 2020. 10.3390/nu12040951. 32235497 10.3390/nu12040951 PMC7231312

[61] D’Souza WN, Douangpanya J, Mu S, Jaeckel P, Zhang M, Maxwell JR, et al. Differing roles for short chain fatty acids and GPR43 agonism in the regulation of intestinal barrier function and immune responses. PLoS ONE. 2017;12(7):e0180190. 10.1371/journal.pone.0180190. 28727837 10.1371/journal.pone.0180190 PMC5519041

[62] Hodgkinson K, El Abbar F, Dobranowski P, Manoogian J, Butcher J, Figeys D, et al. Butyrate’s role in human health and the current progress towards its clinical application to treat gastrointestinal disease. Clin Nutr. 2023;42(2):61–75. 10.1016/j.clnu.2022.10.024. 36502573 10.1016/j.clnu.2022.10.024

[63] Zhang J, Song L, Wang Y, Liu C, Zhang L, Zhu S, et al. Beneficial effect of butyrate-producing Lachnospiraceae on stress-induced visceral hypersensitivity in rats. J Gastroenterol Hepatol. 2019;34(8):1368–76. 10.1111/jgh.14536. 30402954 10.1111/jgh.14536 PMC7379616

[64] Jang H, Lim H, Park KH, Park S, Lee HJ. Changes in plasma choline and the betaine-to-choline ratio in response to 6-month lifestyle intervention are associated with the changes of lipid profiles and intestinal microbiota: the ICAAN study. Nutrients. 2021. 10.3390/nu13114006. 34836260 10.3390/nu13114006 PMC8625635

[65] Lynch JB, Gonzalez EL, Choy K, Faull KF, Jewell T, Arellano A, et al. Gut microbiota Turicibacter strains differentially modify bile acids and host lipids. Nat Commun. 2023;14(1):3669. 10.1038/s41467-023-39403-7. 37339963 10.1038/s41467-023-39403-7 PMC10281990

[66] Arnoriaga-Rodriguez M, Mayneris-Perxachs J, A. Buroka A, Contreras-Rodriguez O, Blasco G, Coll C, Biarnes C, Miranda-Olivos R, Latorre J, Moreno-Navarrete JM, Castells-Nobau A, Sabater M, Palomo-Buitrago ME, Puig J, Pedraza S, Gich J, Perez-Brocal V, Ricart W, Moya A, Fernandez-Real X, Ramio-Torrenta L, Pamplona R, Sol J, Jove M, Portero-Otin M, Maldonado R, Fernandez-Real JM. Obesity impairs short-term and working memory through gut microbial metabolism of aromatic amino acids. Cell Metab. 2020;32(4): 548–560 e7. 10.1016/j.cmet.2020.09.002. 10.1016/j.cmet.2020.09.002 33027674

[67] Zhao J, Liu P, Wu Y, Guo P, Liu L, Ma N, et al. Dietary fiber increases butyrate-producing bacteria and improves the growth performance of weaned piglets. J Agric Food Chem. 2018;66(30):7995–8004. 10.1021/acs.jafc.8b02545. 29986139 10.1021/acs.jafc.8b02545

[68] Mishra SP, Jain S, Wang B, Wang S, Miller BC, Lee JY, et al. Abnormalities in microbiota/butyrate/FFAR3 signaling in aging gut impair brain function. JCI Insight. 2024. 10.1172/jci.insight.168443. 38329121 10.1172/jci.insight.168443 PMC10967378

[69] Huang R, Zhang J, Sun M, Xu L, Kuang H, Xu C, et al. Oat β-glucan enhances gut barrier function and maintains intestinal homeostasis in naturally aging mice. Int J Biol Macromol. 2025;305:141129. 10.1016/j.ijbiomac.2025.141129. 39961571 10.1016/j.ijbiomac.2025.141129

[70] Jaeger JW, Brandt A, Gui W, Yergaliyev T, Hernández-Arriaga A, Muthu MM, et al. Microbiota modulation by dietary oat beta-glucan prevents steatotic liver disease progression. JHEP Reports. 2024;6(3):100987. 10.1016/j.jhepr.2023.100987. 38328439 10.1016/j.jhepr.2023.100987 PMC10844974

[71] Brandt A, Hernández-Arriaga A, Yergaliyev T, Nier A, Halilbasic E, Trauner M, et al. Short-term intake of fiber-rich oat bran but not spelt bran flake mix lowers bacterial endotoxin levels and improves health parameters in healthy, normal weight, young to middle-aged women. J Funct Foods. 2024;112:105929. 10.1016/j.jff.2023.105929.

[72] Cai N, Wu Y, Huang Y. Induction of accelerated aging in a mouse model. Cells. 2022. 10.3390/cells11091418. 35563724 10.3390/cells11091418 PMC9102583

[73] Perlman RL. Mouse models of human disease: An evolutionary perspective. Evolution, Medicine, and Public Health. 2016;2016(1):170–6. 10.1093/emph/eow014. 27121451 10.1093/emph/eow014 PMC4875775

[74] Voetmann LM, Rolin B, Kirk RK, Pyke C, Hansen AK. The intestinal permeability marker FITC-dextran 4kDa should be dosed according to lean body mass in obese mice. Nutr Diabetes. 2023;13(1):1. 10.1038/s41387-022-00230-2. 36604407 10.1038/s41387-022-00230-2 PMC9816099

