# Supplementary material for: Aging-related decline in the liver and brain is accelerated by refined diet consumption
Source: GeroScience. 2025 Sep 26;48(3):4275–94. doi: 10.1007/s11357-025-01897-y (PMC13355980; doi:10.1007/s11357-025-01897-y)
Supplement: Supplementary file 1 — Supplementary file1 (PDF 54.4 KB) [file 11357_2025_1897_MOESM1_ESM.pdf]

Supportive Information

Supplemental Table 1: Composition of diets (modified AIN93G, Ssniff GmbH)

Ingredient		Refined diet	+ cellulose	+ β-glucan
**Casein**	%	20	20	20
**L-Cysteine**	%	0.3	0.3	0.3
**Corn starch**	%	39.2	36.7	34.2
**Maltodextrin**	%	13.2	13.2	13.2
**Sucrose**	%	10	10	10
**Cellulose**	%	5	7.5	-
**β-glucan preparation***	%	-	-	9.96
**Vitamin premix, Ssniff**	%	1	1	1
**Mineral mix, mod. AIN93G**	%	4	4	4
**Choline Cl**	%	0.3	0.3	0.3
**tBHQ (antioxidant)**	%	0.0014	0.0014	0.0014
**Soybean oil**	%	7	7	7
**Proximate content**				
**Crude protein, CP**	% wt/wt	17.7	17.7	17.7
**Crude fat, CL**	% wt/wt	7.1	7.1	7.1
**Crude fibre**	% wt/wt	5.0	7.5	7.5
**Crude ash**	% wt/wt	3.6	3.6	3.6
**Energy (Atwater)**	MJ/kg	16.1	15.7	15.7
**CP**	kcal%	18	19	19
**CL**	kcal%	17	17	17
**Carbohydrate**	kcal%	65	64	64

* containing 75.5% oat beta glucan.
